# Supplementary material for: Machinability enhancement of heat-treated Incoloy 800H during turning using laser-textured carbide inserts under semi-solid MoS₂ lubrication
Source: PLoS One. 2026 May 12;21(5):e0349106. doi: 10.1371/journal.pone.0349106 (PMC13166899; doi:10.1371/journal.pone.0349106)
Supplement: S1 Data — (PDF) [file pone.0349106.s001.pdf]

Supporting Information

S1_Data. Raw experimental dataset for machinability evaluation of heat-treated Incoloy 800H using textured cutting inserts

This supporting file contains the experimental values used to generate Tables 3 and 4 and all corresponding figures in the manuscript.

Table S1. Surface roughness (Ra, µm)

Exp. No.	NT	PT	ET	SCT
1	0.67	0.54	0.77	1.09
2	0.78	0.74	0.98	1.03
3	1.03	1	1.23	1.25
4	0.44	0.4	0.62	0.85
5	0.58	0.41	0.75	0.94
6	0.45	0.44	0.83	1.02
7	0.31	0.3	0.67	1.01
8	0.34	0.32	0.45	0.86
9	0.36	0.34	0.51	1.03

Table S2. Cutting force (Fz, N)

Exp. No.	NT	PT	ET	SCT
1	161.62	142.36	151.23	188.26
2	169.62	148.75	156.24	195.39
3	201.29	187.14	192.34	210.67
4	124.08	122.56	130.79	136.71
5	151.92	139.73	159.41	166.45
6	183.78	158.79	164.86	197.43
7	101.38	91.23	102.16	112.49
8	120.3	111.45	114.68	134.27
9	134.8	120.14	129.47	141.68

Table S3. Cutting power (P, W)

Exp. No.	NT	PT	ET	SCT
1	94.28	83.04	88.22	109.82
2	98.95	86.77	91.14	113.98
3	117.42	109.17	112.2	122.89
4	93.06	91.92	98.09	102.53
5	113.94	104.8	119.56	124.84
6	137.84	119.09	123.65	148.07
7	92.93	83.63	93.65	103.12
8	110.28	102.16	105.12	123.08
9	123.57	110.13	118.68	129.87

Table S4. Specific energy consumption (SEC, J/mm³)

Exp. No.	NT	PT	ET	SCT
1	16.17	14.24	15.13	18.84
2	8.48	7.44	7.81	9.77
3	6.71	6.24	6.41	7.02
4	12.41	12.26	13.08	13.67
5	7.6	6.99	7.97	8.32
6	6.13	5.29	5.5	6.58
7	10.13	9.12	10.21	11.25
8	6.02	5.57	5.73	6.71
9	4.49	4	4.32	4.72

Table S5. Specific cutting pressure (SCPR, N/mm²)

Exp. No.	NT	PT	ET	SCT
1	16162	14236	15123	18826
2	8481	7437.5	7812	9769.5
3	6709.6	6238	6411.3	7022.3
4	12408	12256	13079	13671
5	7596	6986.5	7970.5	8322.5
6	6126	5293	5495.3	6581
7	10138	9123	10216	11249
8	6015	5572.5	5734	6713.5
9	4493.3	4004.6	4315.6	4722.6

Table S6. Tool-tip temperature (°C)

Exp. No.	NT	PT	ET	SCT
1	88.6	75.7	80.2	92.4
2	92.7	78.1	83.7	96.8
3	95.4	81.5	87.3	100.8
4	119.5	97.3	108.6	112.8
5	123.3	105.9	117.9	121.9
6	125.3	73.2	81.7	88.6
7	88.3	78.2	86.7	93.6
8	113.6	92.3	97.6	101.2
9	121.6	99.7	111.3	116.9

Table S7. Material removal rate (MRR, mm^3/s)

Exp. No.	MRR
1	5.83
2	11.67
3	17.5
4	7.5
5	15
6	22.5
7	9.17
8	18.33
9	27.5
