# Supplementary material for: Machinability enhancement of heat-treated Incoloy 800H during turning using laser-textured carbide inserts under semi-solid MoS₂ lubrication
Source: PLoS One. 2026 May 12;21(5):e0349106. doi: 10.1371/journal.pone.0349106 (PMC13166899; doi:10.1371/journal.pone.0349106)
Supplement: Attachment — Submitted filename: Response to Reviewers.pdf [file pone.0349106.s003.pdf]

Manuscript Title:

Machinability enhancement of heat-treated Incoloy 800H during turning using laser-textured carbide inserts under semi-solid MoS_2 lubrication

Manuscript ID: PONE-D-26-12246

Dear Academic Editor and Reviewers,

We sincerely thank the Academic Editor and the Reviewers for their careful evaluation of our manuscript and for their constructive and insightful comments. We greatly appreciate the time and effort invested in reviewing our work. The suggestions have helped us significantly improve the clarity, technical rigor, completeness, and presentation quality of the manuscript. All comments have been carefully addressed in the revised version. Corresponding revisions have been incorporated in the manuscript and highlighted in the red colour. A detailed point-by-point response is provided below.

Responses to Academic Editor

Comment

Please ensure that your manuscript meets PLOS ONE's style requirements, including those for file naming.

Response

We thank the Academic Editor for this important suggestion. The manuscript has been carefully revised to comply fully with PLOS ONE formatting requirements. Specifically:

- figure citations formatted as “Fig 1”, “Fig 2”, etc.
- tables cited sequentially in order of appearance
- headings revised in sentence-case format
- figure captions retained within manuscript body
- figures uploaded separately as individual TIFF files
- supporting information caption added after references
- file naming conventions updated according to journal guidelines

These revisions ensure full compliance with journal formatting standards.

Comment

Please confirm whether your submission contains all raw data required to replicate the results of your study.

Response

We thank the Academic Editor for emphasizing reproducibility requirements. The complete minimal dataset underlying all results presented in the manuscript has now been provided as Supporting Information file **S1_Data**.

This dataset includes:

- surface roughness values
- cutting force measurements
- cutting power measurements
- specific cutting pressure
- specific energy consumption
- tool-tip temperature
- material removal rate

These data correspond directly to the values used to construct all tables, plots, and statistical analyses reported in the manuscript. Therefore, the submission fully complies with the PLOS ONE data availability policy.

Comment

We notice that your supplementary figures are uploaded with the file type 'Figure'. Please amend the file type to 'Supporting Information'.

Response

We thank the Academic Editor for identifying this issue. The supporting dataset has now been correctly uploaded as Supporting Information (S1_Data). No supplementary figures are included in the submission. All primary figures are provided separately as individual figure files according to journal submission requirements.

Comment

If the reviewer comments include a recommendation to cite specific previously published works, please review and evaluate these publications to determine whether they are relevant and should be cited.

Response

We thank the Academic Editor for this suggestion. The references recommended by Reviewer 2 have been carefully evaluated and incorporated into the revised manuscript where relevant. These citations strengthen the discussion related to hybrid lubrication strategies and textured cutting tool performance during machining of nickel-based superalloys.

Comment

The authors must incorporate the suggestions provided by the reviewers in the revised manuscript.

Response

We respectfully confirm that all reviewer comments have been carefully addressed. In particular, the manuscript has been improved by:

- adding recent literature in the Introduction
- clarifying research objective and novelty
- including detailed laser texturing parameters
- reporting texture geometry dimensions
- expanding MoS_2 lubrication description
- clarifying infrared temperature measurement methodology
- adding experimental repeatability analysis
- incorporating ANOVA-based statistical evaluation
- summarizing factor significance in Table 3
- revising Conclusions in structured format
- adding percentage performance improvements
- providing Supporting Information dataset (S1_Data)

These revisions substantially improve technical clarity and reproducibility.

<u>**Responses to Reviewer 1**</u>

Comment 1

Include more recent articles in the introduction section

Response

We sincerely thank the Reviewer for this helpful suggestion. Additional recent literature related to laser surface texturing, hybrid lubrication strategies, and machinability enhancement of nickel-based superalloys has been incorporated into the Introduction section to improve the contextual relevance and strengthen the scientific background of the study.

Comment 2

The objective and novelty in the research should be included in the last paragraph of the introduction section.

Response

We thank the Reviewer for this valuable suggestion. The final paragraph of the Introduction has been revised to clearly state the research objective and explicitly highlight the novelty of the present work, particularly the combined investigation of laser-textured carbide inserts and semi-solid MoS_2 lubrication under heat-treated Incoloy 800H machining conditions, which has not been systematically reported in earlier studies.

Comment 3

Include the laser texture parameters selected

Response

We thank the Reviewer for this important suggestion. The Materials and Methods section has been revised to include complete laser surface texturing parameters, including wavelength (1064 nm), pulse frequency (20 kHz), average power (18 W), scanning speed (300 mm/s), groove width (120 µm), texture spacing (250 µm), texture depth (35 µm), and offset distance (150 µm) from the cutting edge. These additions improve experimental transparency and ensure reproducibility of the texturing process.

Comment 4

Include the dimensions of the textured surfaces on the rake surface.

Response

We thank the Reviewer for this useful suggestion. The geometric characteristics of the fabricated textures, including groove width, spacing, depth, and offset distance from the cutting edge, have now been explicitly reported in the Materials and Methods section to improve clarity and facilitate reproducibility of the experimental methodology.

Comment 5

Include citation to validate this statement

Response

We thank the Reviewer for this valuable suggestion. Appropriate supporting references have now been incorporated to validate the role of parallel micro-textures in improving lubricant retention and reducing tool–chip contact area, thereby decreasing friction and improving surface quality during machining of nickel-based superalloys.

Comment 6

Generally nickel based alloys has poor thermal conductivity. Hence, the highest temperature recorded were 125°C. It is not possible. Check this

Response

We sincerely thank the Reviewer for this important observation. The manuscript has been revised to clarify that the reported temperatures correspond to localized external rake-face surface temperatures measured using infrared sensing near the tool–chip interface. These values therefore represent surface temperatures rather than peak shear-zone temperatures, which are typically significantly higher and may exceed 600 °C during machining of nickel-based superalloys. This clarification has now been incorporated into the Results and Discussion section with supporting references to improve interpretation of the temperature measurements.

Comment 7

Results and discussion section- include more citations to validate your findings.

Response

We thank the Reviewer for this helpful suggestion. Additional relevant references have now been incorporated throughout the Results and Discussion section to support the interpretation of observed trends in surface roughness, cutting force, tool-tip temperature, and specific energy consumption, thereby strengthening the scientific justification of the reported findings.

Comment 8

Rewrite the conclusion in a point wise.

Response

We thank the Reviewer for this valuable suggestion. The Conclusions section has been completely revised and reorganized into a structured pointwise format to improve clarity, readability, and accessibility of the key outcomes of the study.

Comment 9

Conclusion – include the % wise comparison of your findings.

Response

We thank the Reviewer for this useful suggestion. Percentage improvements in surface roughness, cutting force, tool-tip temperature, and specific energy consumption obtained using the parallel-textured insert have now been incorporated into the Conclusions section to better highlight the quantitative performance advantages achieved in the present investigation.

Responses to Reviewer 2

We sincerely thank the Reviewer for the encouraging assessment of the manuscript and for the constructive suggestions that helped improve the technical clarity and reproducibility of the study. All comments have been carefully addressed as detailed below.

Comment

Forged rods of Incoloy 800H superalloy with a diameter of 30 mm and length of 120 mm were used as the work material. how dimesion is fixed?

Response

We thank the Reviewer for this important observation regarding specimen geometry selection. The manuscript has been revised to clarify that the selected dimensions (30 mm diameter and 120 mm length) correspond to standard cylindrical turning specimen geometry commonly adopted in machinability investigations of nickel-based superalloys. These dimensions ensure:

- sufficient machining length for achieving steady-state cutting conditions,
- elimination of transient tool entry/exit effects during measurement,
- reliable acquisition of force, temperature, and surface roughness data,
- repeatability across multiple experimental trials.

A clarifying statement with supporting literature references from comparable machinability investigations on nickel-based superalloys have now been incorporated in the Materials and Methods section to justify the selected specimen geometry and ensure consistency with established experimental practice.

Comment

Table 1. Chemical composition of Incoloy 800H (wt %). provide citation.

Response

We thank the Reviewer for this useful suggestion. A supporting reference consistent with ASTM B407 standard specifications for Incoloy 800H has now been added below Table 1 to validate the reported chemical composition obtained from spectroscopy analysis. The composition values are consistent with ASTM standard specifications for Incoloy 800H and were experimentally verified using spectroscopy analysis. The corresponding citation has been included in the revised manuscript immediately below Table 1.

Comment

Table 2. Experimental design layout for the turning experiments indicating cutting speed (Vc), feed rate (f), and depth of cut (ap). how the parameters are selected? Why Depth of cut (ap) (mm) is constant? Which design of experiment has been adopted?

Response

We sincerely thank the Reviewer for highlighting the need to clarify the experimental design methodology. The manuscript has been revised to provide a detailed explanation below Table 2.

Specifically:

- machining parameter ranges were selected based on recommended turning conditions for nickel-based superalloys reported in prior literature,
- both low- and moderate-speed regimes relevant to industrial machining practice were considered,
- a full factorial experimental design (3 × 3) was adopted to evaluate the individual and interaction effects of cutting speed and feed rate,
- the depth of cut was maintained constant at 1 mm to isolate the influence of the primary control parameters while avoiding excessive variation in tool loading and thermal conditions.

In addition, statistical evaluation of parameter significance using ANOVA at a 95% confidence level has now been included and summarized in Table 3, further confirming the adequacy and robustness of the selected experimental design. The inclusion of ANOVA further confirms the statistical adequacy of the selected factorial design for evaluating both the primary effects and interaction effects of machining parameters on the measured machinability indicators.

Comment

How texturing ids done?

Response

We thank the Reviewer for requesting clarification regarding the surface texturing methodology. The Materials and Methods section has been expanded to provide a detailed description of the laser surface texturing process.

The revised manuscript now specifies:

- Nd:YAG laser wavelength: 1064 nm
- pulse frequency: 20 kHz
- average power: 18 W
- scanning speed: 300 mm/s
- groove width: 120 µm
- groove spacing: 250 µm
- texture depth: 35 µm
- offset distance from cutting edge: approximately 150 µm

These parameters ensured precise fabrication of micro-textures without inducing thermal damage near the cutting edge and allowed effective interaction between the textured region and the tool–chip contact interface during machining.

Comment

Conclusions may be stated point wise.

Response

We thank the Reviewer for this valuable suggestion. The Conclusions section has been completely revised and reorganized into a structured pointwise format to improve readability and clearly highlight the principal findings of the study.

Comment

Refer following papers

Response

We sincerely thank the Reviewer for recommending these relevant and valuable references. These publications were carefully reviewed and incorporated into the revised manuscript where appropriate to strengthen the discussion on hybrid lubrication strategies and the tribological advantages of textured cutting tools during machining of difficult-to-machine superalloys.

Their inclusion improves the contextual positioning of the present work within current developments in sustainable machining research.

Responses to Reviewer 3

We sincerely thank the Reviewer for the careful evaluation of the manuscript and for the insightful technical suggestions. These comments helped us substantially improve the experimental clarity, statistical robustness, and physical interpretation of machining behaviour reported in the study.

Comment

How often were the individual cutting tests repeated? Are the values shown average or maximum? What was the dispersion of values?

Response

We thank the Reviewer for emphasizing the importance of experimental repeatability and uncertainty reporting. The manuscript has been revised to clarify that each machining experiment was conducted three times under identical cutting conditions, and the reported values correspond to the average of the measured responses.

In addition, the standard deviation of repeated measurements remained within ±4% of the mean values, confirming good repeatability and reliability of the experimental observations. This information has now been explicitly included in the Materials and Methods section.

Comment

What was the depth of the textures? After how long of cutting time did the cutting tool wear greater than the depth of the applied texture?

Response

We thank the Reviewer for this important observation regarding the functional durability of the micro-textures. The texture depth has now been explicitly reported as 35 μm in the Materials and Methods section.

Furthermore, crater wear observed during the experimental cutting duration remained lower than the texture depth, ensuring that the lubricant-retention capability of the textured surfaces was maintained throughout the machining trials. This clarification has now been incorporated in the revised manuscript.

Comment

At what distance from the cutting edge was the texture of PT (Fig. 3a), and was it in the range of cutting depth ap = 1 mm?

Response

We thank the Reviewer for requesting clarification regarding texture positioning relative to the cutting edge. The manuscript now specifies that the textures were located at an offset distance of approximately 150 µm from the cutting edge, ensuring effective interaction with the tool–chip contact region while preserving cutting edge strength.

Since the cutting depth was maintained at 1 mm, the textured region remained fully within the active tool–chip contact zone during machining operations. This clarification has been added to the Materials and Methods section.

Comment

MoS_2 lubrication description should be extended.

Response

We thank the Reviewer for this valuable suggestion. The description of semi-solid MoS_2 lubrication has been expanded to include its lamellar crystal structure, low interlayer shear strength, and ability to form a stable tribofilm under boundary lubrication conditions typical of machining nickel-based superalloys. The role of MoS_2 in reducing friction and improving thermal stability at the tool–chip interface is now discussed in greater detail in the Materials and Methods section.

Comment

The description of the temperature measurements requires further clarification – how exactly were the measurements performed? Were the tool temperature measured, or the temperature of the chip formed on the rake face?

Response

We sincerely thank the Reviewer for highlighting the need for clarification. The manuscript now explicitly states that temperature measurements were obtained using an infrared thermometer positioned toward the external rake-face region adjacent to the tool–chip interface.

It is also clarified that these values represent localized surface temperatures of the cutting tool rather than peak temperatures within the primary shear deformation zone, which are typically significantly higher during machining of nickel-based superalloys. This clarification has been incorporated in the Results and Discussion section.

Comment

Fig. 10 b – the authors should explain the reason for the increase and then decrease in temperature for vc = 45 m/min and vc = 55 m/min, respectively.

Response

We thank the Reviewer for this insightful observation. An explanation has now been added in the Results and Discussion section indicating that the initial increase in temperature with cutting speed is associated with increased frictional heat generation, whereas the subsequent reduction at higher cutting speed is attributed to reduced tool–chip contact time, enhanced thermal softening of the workpiece material, and improved sliding behaviour facilitated by semi-solid MoS_2 lubrication.

Comment

The authors did not present a statistical analysis of the results of the experimental research…

Response

We sincerely thank the Reviewer for emphasizing the importance of statistical validation. In response, statistical evaluation using analysis of variance (ANOVA) at a 95% confidence level has now been incorporated into the manuscript.

A summary of factor significance has been included as Table 3, demonstrating:

- dominant influence of feed rate on surface roughness,
- significant influence of cutting speed on cutting force and tool-tip temperature,
- interaction effects between cutting speed and feed rate on specific energy consumption and specific cutting pressure.

These additions strengthen the statistical rigor of the study, quantitatively establish the relative influence of machining parameters on each response variable, and directly address the Reviewer's concern regarding the absence of statistical validation in the original submission.

We sincerely appreciate the constructive comments and thoughtful recommendations provided by the Academic Editor and Reviewers. The manuscript has been substantially improved in terms of methodological clarity, statistical validation, experimental reproducibility, and technical interpretation as a result of these suggestions. We respectfully hope that the revised version meets the expectations of the journal and is now suitable for publication in PLOS ONE.
